# A novel computational signal processing framework towards multimodal vital signs extraction using neck-worn wearable devices

**DOI:** 10.1038/s41598-024-72184-7

**Published:** 2024-09-27

**Authors:** Rawan S. Abdulsadig, Esther Rodriguez-Villegas

**Affiliations:** Wearable Technologies Lab, Department of Electrical and Electronic Engineering, Imperial College London, London, SW7 2BT UK

**Keywords:** Vital signs, Wearable devices, Neck, Photoplethysmography (PPG), Accelerometer (Acc), Exponentially weighted moving average (EWMA), Data processing, Computational science, Biomedical engineering

## Abstract

Pulse rate (PR) and respiratory rate (RR) are two of the most important vital signs. Monitoring them would benefit from easy-to-use technologies. Hence, wearable devices would, in principle, be ideal candidates for such systems. The neck, although highly susceptible to artifacts, presents an attractive location for a diverse pool of physiological biomarkers monitoring purposes such as airflow sensing in a non-obstructive manner. This paper presents a methodology for PR and RR estimation using photoplethysmography (PPG) and accelerometry (Acc) sensors placed on the neck. Neck PPG and Acc signals were recorded from 22 healthy participants for RR estimation, where the resting subjects performed guided breathing following a visual metronome. Neck PPG signals were obtained from 16 healthy participants who breathed through an altitude generator machine in order to acquire a wider range of PR readings while at rest. The proposed methodology was able to provide rate estimates via a combination of recursive FFT-based dominance scoring coupled with an exponentially weighted moving average (EWMA)-driven aggregation scheme. The recursion aimed at bypassing sudden intra-window amplitude deviations caused by momentary artifacts, while the EWMA-based aggregation was utilized for handling inter-window artifact-induced deviations. To further improve estimation stability and confidence, estimates were calculated in the form of rate bands taking into account the relevant clinically acceptable error margins, and results when considering rate values and rate bands are presented and discussed. The framework was able to achieve an overall pulse rate value accuracy of $93.67\pm 7.64$% within the clinically acceptable ± 5 BPM with reference to the gold-standard reference devices while providing an overall respiratory rate value accuracy within the clinically appropriate ± 3 BrPM of $94.94\pm 3.56$% with reference to the guiding visual metronome, and $88.4\pm 7.63$% with respect to the gold-standard reference device. The proposed methodology achieves acceptable PR and RR estimation capabilities, even when signals are acquired from an unusual location such as the neck. This work introduces novel ideas that can lead to the development of medical device outputs for PR and RR monitoring, especially capitalizing on the advantages of the neck as a multi-modal physiological monitoring location.

## Introduction

The early detection of potential cardiovascular and respiratory illnesses can be enabled by the continuous monitoring of vital signs such as pulse rate (PR) and respiratory rate (RR). Continuous monitoring of these signs can also help in the evaluation of the efficacy of relevant medical interventions^1,2^. In the context of preventing sudden unexpected death in patients with epilepsy (SUDEP), integrating multiple vital signs such as PR and RR along with blood oxygen saturation (SpO2) as well as sleep posture can provide a comprehensive view that could allow for the prevention of such tragic events. A retrospective study (MORTEMUS) that comprehensively evaluated data obtained from various epilepsy monitoring units has shown that the main mechanism leading to SUDEP starts with an early severe instability of both respiratory and cardiac functions after generalized tonic-clonic seizures, which might lead to immediate death or delayed terminal cardiorespiratory arrest after several minutes of unstable cardiorespiratory function that is aggravated by profound hypoxia, and potentially promoted by being at prone position^3^. A reliable real-time monitoring system that can analyze trends and patterns in PR and RR could enable timely interventions when such abnormalities occur, potentially saving lives.

Electrocardiogram (ECG) is the gold standard for monitoring the electrical activity of the heart, from which parameters of clinical relevance can be obtained, such as heart rate which is calculated by measuring the time interval between successive R-waves (peaks of the QRS complex) in the ECG waveform, while Capnography is the gold standard for monitoring respiratory rate by means of measuring the concentration of CO2 in respiratory gases. ECG signals which capture the electrical activity of the heart muscle are acquired typically via multiple electrodes placed on the chest area, while Capnography requires the patient to breathe into an oral/nasal cannula that captures the exhaled air, and thereafter measures the frequency of breathing. The special setup and application of ECG electrodes, although feasible in clinical environments, can be impractical in domestic settings^4^, while the measurement accuracy of the Capnograph is highly dependent on adequate cannula positioning, which can be compromised with any head movements, making it impractical for most patients, and most definitely for domestic use. Due to the impracticality of Capnography, respiratory rate, in particular, is often overlooked in typical clinical practice, being manually counted and registered once every 8-10 hours at best, due to the lack of medical staff time needed to consistently and accurately count and monitor the breathing rate of their patients^5^.

Wearable as well as non-contact monitoring technologies can be the best solution to the aforementioned usability and practicality issues. Amongst the available non-contact methods including optical vibro-cardiography, infrared thermography, ultrasound and Doppler radars^6–9^; cameras gained significantly growing interest in recent years due to their lower cost and convenience as well as the considerable advancements in image and video processing using deep learning techniques^10^. Using cameras, however, suffers from severe reliability issues due to region of interest (ROI) loss and drift caused by motion variance, illumination variance, as well as change in the distance from the camera^11^. Furthermore, using cameras for healthcare monitoring can raise some legitimate privacy concerns, especially when they are operating in the visible spectrum^10,12^. Although this issue can be bypassed by their deployment in public spaces where surveillance is already present, however, their use in homes for bedside monitoring, as is the case for sudden unexpected death in epilepsy (SUDEP) where monitoring of vital signs is especially needed during sleep, can be unsuitable. Therefore, contact sensors implemented in wearable form are more favorable in these scenarios.

Wearable sensors offer unique opportunities for providing convenient and easy-to-use devices that encourage user adherence, however, they often provide suboptimal accuracies compared to traditional clinical methods for vital sign measurements, and many studies lack the clear evaluation against clinically validated measurement systems^13–15^. Furthermore, many studies conduct very short experiments (less than 5 min) on a small number of subjects, compromising the statistical power of the results and limiting generalizability. Therefore, there is wide room for improvement when it comes to vital signs extraction using wearable sensors intended for clinical use.

**Fig. 1:**
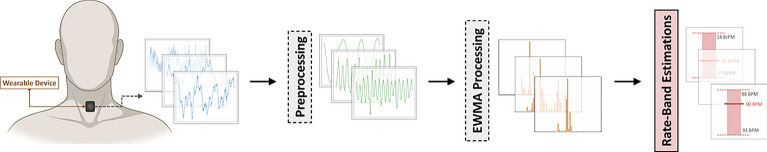
Overview of the methodology (with illustrative examples).

In the context of pulse rate monitoring; Photoplethysmography (PPG) is often preferred as a substitution for traditional ECG signals since it optically measures volumetric changes in blood vessels^16^ correlating with heart beats, whilst being more convenient and lower in cost, making it more suitable for everyday use^17^. PPG signals can also be strongly modulated by breathing rate when acquired from the neck^18–20^, making the placement of the sensor on the neck an advantageous setup for both PR and RR monitoring. PR and RR estimation using acoustic sensors placed on the neck^5,21^ as well as inertial sensors such as accelerometers (Acc) placed on the chest area were presented in previous work^22,23^.

Most studies utilize peak identification and verification techniques in order to measure PR or RR, either in time or frequency domain^16,22,24^, which can suffer from grave errors when signals are acquired from the neck where signals are often superimposed by intrinsic as well as external physiological noise types that are specific to that location^18,19^. Furthermore, the neck is especially challenging when working with PPG signals due to the rich network of major blood vessels including the jugular veins^25^. This could result in complex and inconsistent waveforms, limiting the transferability of signal processing approaches applied to other locations, and making conventional techniques for obtaining vital signs (especially from the finger) highly unreliable when applied to neck signals.

This work presents a novel computational signal processing framework that can be used for PR and RR estimation from photoplethysmography (PPG) and accelerometer (Acc) signals acquired from the neck, using a combination of recursive FFT-based dominance frequency scoring and exponentially weighted moving average (EWMA)-driven aggregation which provides the final estimates in the form of rate values or rate-bands. The term ’Estimation’ refers to the process of indirectly deriving the measurements of the physiological metrics of concern using non-invasive means, in this work via wearable sensors, integrated with mathematical computations and algorithms. Although this work is anticipated to be extendable to various scenarios, this study focused on monitoring during sleep. Figure 1 shows a high-level illustration of the methodology presented in this work.

## Methods

### Data acquisition

Photoplethysmography (PPG) data was obtained using a reflectance PPG sensor (MAX30102, MAXIM integrated) that emits red (650–670 nm) and infrared (870–900 nm) light, while accelerometer (Acc) data was acquired via a triaxial accelerometer (LIS2DH12, ST Electronics). Both sensors were integrated with an NRF5232 microcontroller (Nordic Semiconductor) along with a rechargeable 3.8V 80 mAh lithium polymer battery, all of which were housed in an additive 3D-printed enclosure constituting the wearable device shown in Fig. 1. The PPG signals were sampled at 400 Hz while the Acc signals were sampled at 100 Hz. The data was then transmitted wirelessly via Bluetooth Low Energy (BLE) to a customized data acquisition iOS app. When conducting the data acquisition sessions, this wearable device was positioned approximately 1 inch above the suprasternal notch on the neck using a double-sided adhesive shaped in a way not to obstruct the PPG light trajectory.

The experiments included in this work were approved by the Local Ethics Committee of Imperial College London (ICREC reference number: 18IC4358). Assuming an effect size d = 0.2 (based on Cohen’s suggestion for small detectable differences), a minimum output rate of 0.5 Hz, and a minimum duration of 30 s per each measured rate, it was determined using a paired two-sided t-test that the required number of subjects was 14 in order to achieve 80% statistical power at 5% significance level. Healthy subjects without any known respiratory or cardiac conditions, between the age of 18 and 70 years old were targeted for this work. Calls for participation were distributed via email and social means. All experiments in this work were performed in accordance with the Declaration of Helsinki. All recruited participants were asked to sign an informed consent form before noting their basic demographic information and starting their session, and all sensors were handled and attached to the participants by the study conductor and cleaned appropriately afterward.

#### Respiratory rate experiment

22 healthy participants were recruited for this experiment (15 males and 7 females), with an average age of $28 \pm 5.26$ years old and an average BMI of $23.7 \pm 3.89$ (kg/m$^{2}$).

**Fig. 2:**
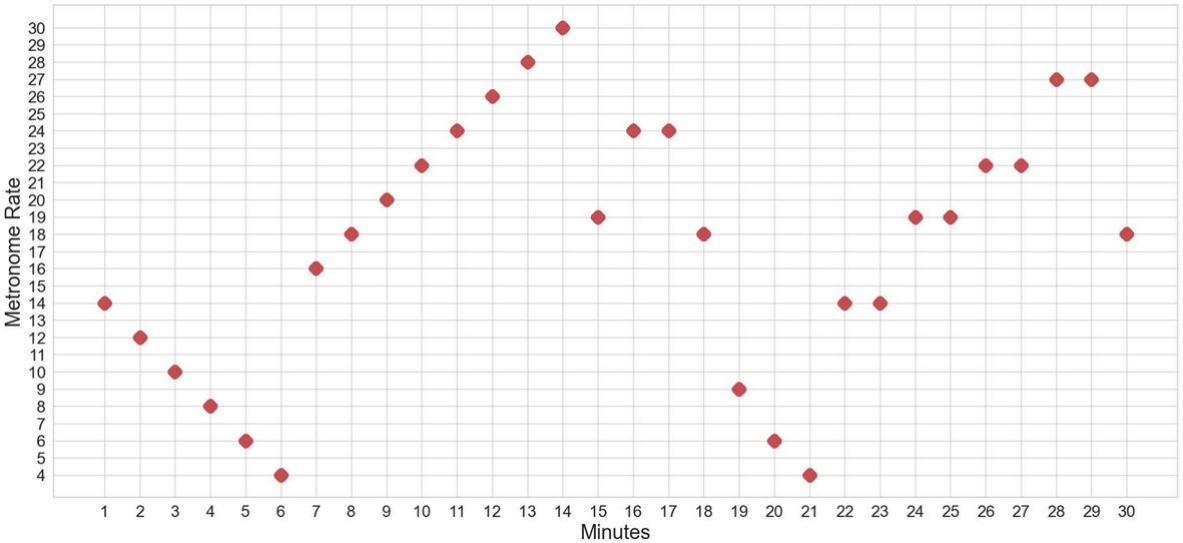
The progression of the experimental protocol showing the breathing rate changes guided by the visual metronome over time.

Participants rested in the supine position, and an oral/nasal cannula and a finger probe connected to the ground truth reference device Capnostream™35 (Medtronics) were attached to them. In this experiment, participants were directed to follow a visual metronome to guide their breathing rate. The visual metronome was designed as a pulsating breathing circle that expanded to resemble inhalation and contracted to resemble exhalation. The guiding breathing rates ranged between 4 and 27 BrPM, where each rate was retained for at least 1 min. The study protocol consisted of gradual 2-BrPM increments and decrements from 14 to 27 BrPM and from 14 to 4 BrPM, respectively. It also included rate changes at larger steps (up to 5 BrPM) to simulate abrupt breathing rate changes. Each data acquisition session lasted for 40–50 min, providing 30 min worth of data. Both PPG and Acc signals were available in this data acquisition experiment. Figure 2 shows the progression of the experimental protocol.

#### Pulse rate experiment

16 healthy participants conducted this experiment (9 males and 7 females). The average age of the participants was $29 \pm 6.3$ years, and their average BMI was $23.52 \pm 3.7$ (kg/m$^{2}$).

**Fig. 3:**
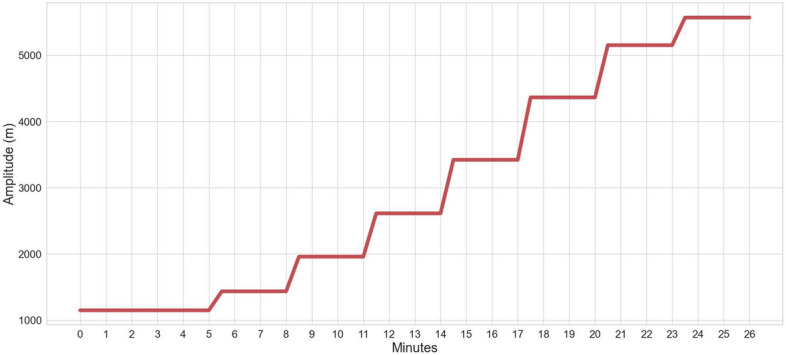
Experimental protocol example showing the simulated altitudes and their minimum durations in the pulse rate experiment.

Each participant was asked to lie down on their back in the supine position, and given an air-cushioned breathing mask connected to an altitude generator machine (Everest Summit Altitude Generator) which was set to gradually reduce the oxygen intake of the participant throughout the session by simulating different altitudes, allowing for a wider range of pulse rate values while being at rest. Each simulated altitude lasted for at least 2.5 min. Two ground truth reference systems were used, namely SOMNOscreen™ plus (SOMNOmedics) and Capnostream™35 (Medtronics), and their respective finger oximeters were attached to the participant’s middle and ring fingers. Each acquisition session was 30–40 min long which provided 26–35 min worth of data, and the overall range of recorded pulse rates was between 45 and 124 BPM. Only PPG signals were available in this data acquisition experiment. Figure 3 overviews the experimental protocol by showing the different altitudes simulated by the altitude generator machine over the experiment.

### Signal preprocessing

**Fig. 4:**
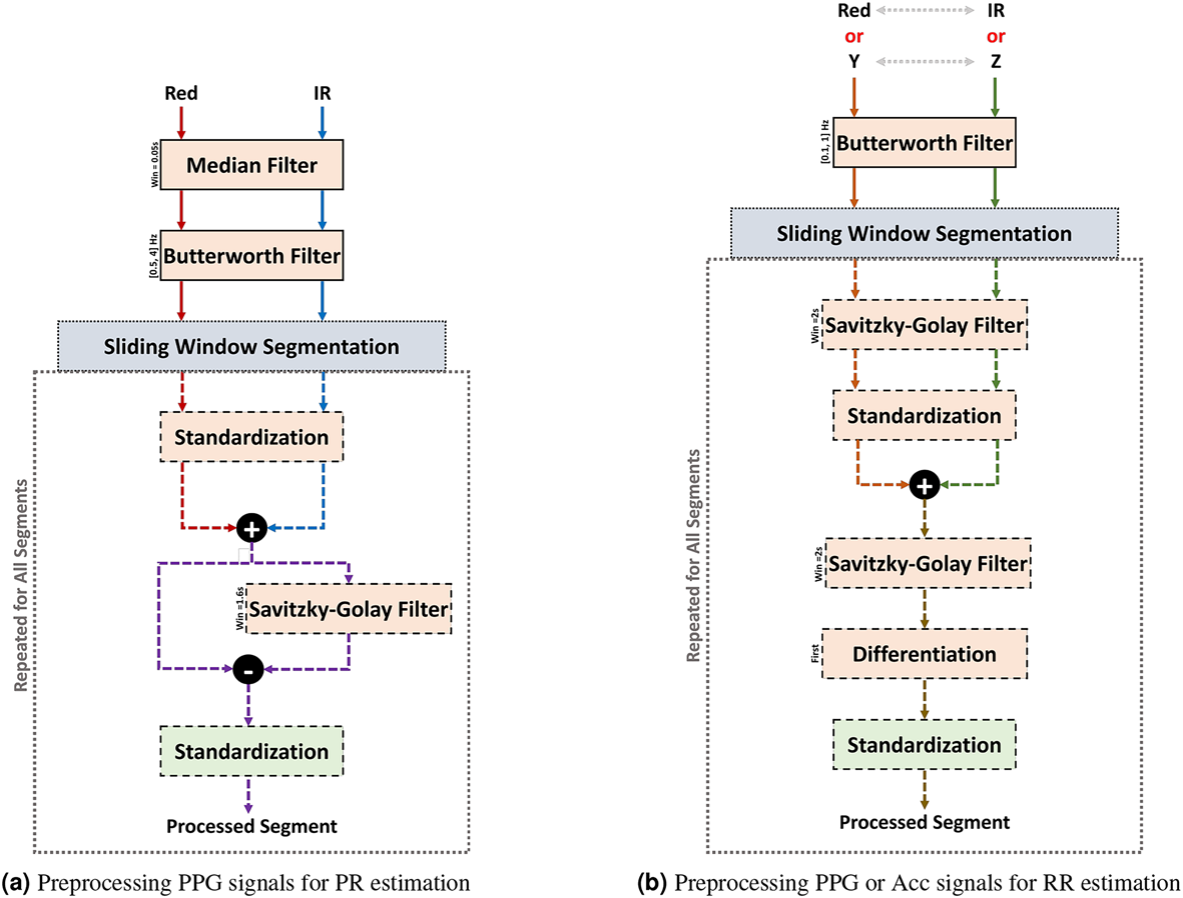
Flow diagrams of the preprocessing steps applied to the (**a**) PPG’s Red and IR channels for pulse rate estimation, and (**b**) PPG’s Red and IR channels or the Acc’s Y and Z channels for respiratory rate estimation].

Signals obtained from the sensors were processed before being used for rate extraction. Due to the nature of the signals and the location of the device, the traditional use of simple band-pass filters without further processing was found to result in poor rate estimation performance in prior trials. To this end, two preprocessing pipelines were formulated for pulse rate extraction and respiratory rate extraction. This is described in the following sub-sections. In both cases, a processing 30-s overlapping sliding window sliding by 1 s was used. Furthermore, producing the processed segments required both Red and IR channels when using the PPG data, and both Y and Z channels when using the Acc data.

#### For pulse rate estimation

Red and IR PPG channels were downsampled to 100 Hz since it was found to be sufficient for acquiring rate estimates. To eliminate transient spiking noise, each of the channels was filtered using a 0.05 s median filter. A 1st-order [0.5, 4] Hz band-pass Butterworth filter was then applied to the signals. The filtered Red and IR segments were standardized then combined using time-wise addition, producing a unified PPG signal representation. To further reduce low-frequency baseline deviations, a 2nd order Savitzky–Golay filter with a window size of 1.6 s was applied to a copy of the PPG segment which was then subtracted from it. This method and window size were chosen empirically following extensive trials. Finally, the filtered signal was standardized. Figure 4a illustrates these steps in a flow diagram.

#### For respiratory rate estimation

When estimating respiratory rate using the PPG’s Red and IR channels or the accelerometer’s Y and Z channels, the same pipeline can be used to obtain the processed segments. The accelerometer’s X channel was not used due to the weak influence of the breathing movement in that dimension given the sensor placement.

As shown in Fig. 4b, each channel’s signal was filtered using a 1st-order [0.1, 1] Hz band-pass Butterworth filter before segmenting it using the sliding window. Once segmented, the signals were smoothed using a 2nd-order 2-s window Savitzky–Golay filter, then standardized before combining them via time-wise addition. The combined signals were then further smoothed using another 2nd-order Savitzky–Golay filter with a window size of 2 s. Baseline deviations were reduced by taking the numerical first derivative of the signal. Finally, the signals were standardized in preparation for the following steps.

### Exponentially weighted moving average (EWMA)-based rate estimation

Algorithm 1– provides a detailed description of how to arrive at a rate-value and rate-band estimate at time t ($RV_t$ and $RB_t$), along with a confidence indication of those estimates, given a processed segment. In this work, rate-value estimates are single absolute values equivalent to what is conventionally produced by monitoring devices, while rate-band estimates give ranges of values that are most likely to contain the true value (within clinically acceptable margins), proposing a non-conventional way of producing rate measurements especially when confidence is low due to irregularities or artifacts. The same main steps can be used for both pulse rate and respiratory rate estimation.

#### Probability estimation

Applying the discrete-Fourier transform (DFT) to a signal gives the frequency-domain representation where the spectral content of the signal is revealed. The observed spectral content can be thought of as the likelihood of the presence of each frequency component given the signal. To this end, the softmax function was applied to the standardized amplitude spectral density of each segment (or sub-segment) in order to pull the high and low values further apart from each other, while ensuring that the sum of all components is equal to 1. This resulted in the predominant frequencies having the highest peaks, while most of the weak frequency components residing below 0.1. This method was developed after extensive empirical trials. The function *GetProbaEstimations* in Algorithm 2 shows these steps in pseudocode. This process was bounded within the frequency range of concern, those ranges were [0, 4] Hz for pulse rate, and [0, 1] Hz for respiratory rate. Frequencies were translated to per-minute rates residing within the rate ranges [0, 240] BPM and [0, 60] BrPM, respectively.

#### Segment-wise dominance score

The purpose of this step was to obtain probability information about the segment while recursively dissecting it into smaller sub-segments as appropriate, and then to acquire dominance scores by aggregating them over the observed rates. The presence of sudden amplitude deviations guided the dissection of segments. This was done by marking areas where the standardized values of a segment (or sub-segment) were deviating beyond the range [$-3$, 3], and only accepting the regions around those areas if they were at least 5 s long. The aggregation of estimates was done by taking the weighted sum of the per segment/sub-segment estimations for each observed rate, using the level of recursion associated with each of them as the weight. Since the rates associated with probability estimation values less than 0.1 were found to be neglectable, the aggregation was conditioned by the values being at least 0.1. Furthermore, the level-based weighting allows the reduced certainty of the FFT-based probability estimations associated with the shortened length of the analyzed signals to be taken into account, since the length was generally associated with the level of recursion in this framework. In other words, the contribution provided by the sub-segment probability estimates to the per-rate dominance scores was inversely proportional to their level of recursion. Recursion was terminated once it exceeded a depth of 20 levels or if the sub-segment was shorter than 5 s. The pseudocode describing function *GetDominanceScores* in Algorithm 2 walks through the detailed implementation of this method, in addition to the function *UpdateDS* in Algorithm 2 which shows the implementation of the aggregation subroutine. In the case of using multiple modalities for rate estimation, as is the case when estimating respiratory rate using PPG and Acc signals in this work, the segment-wise dominance scores would be inferred from each modality independently, then combined in the same manner described in function *UpdateDS*.

**Algorithm 1:**
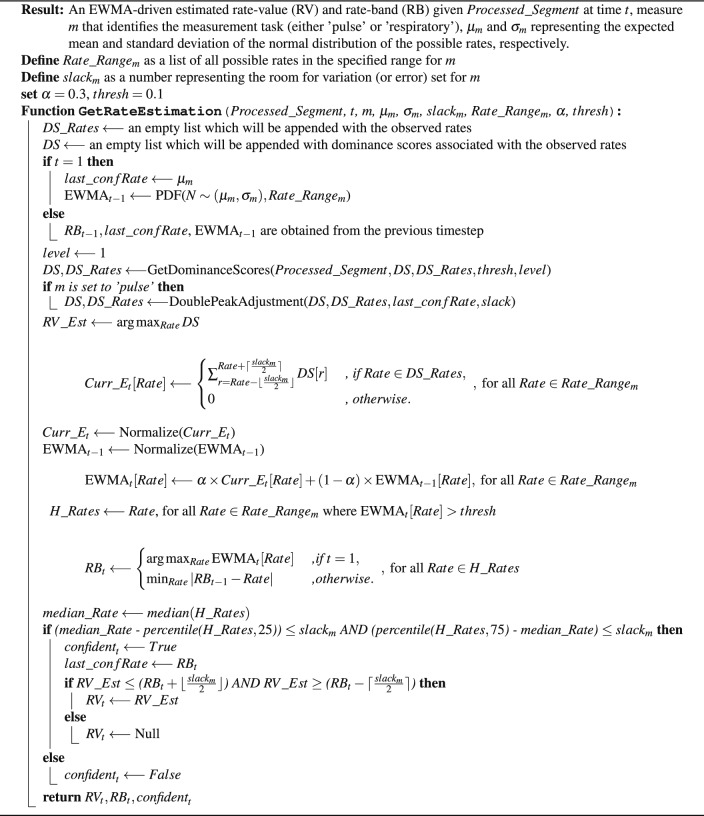
EWMA-driven rate estimation Algorithm, Part 1: Main function.

**Algorithm 2:**
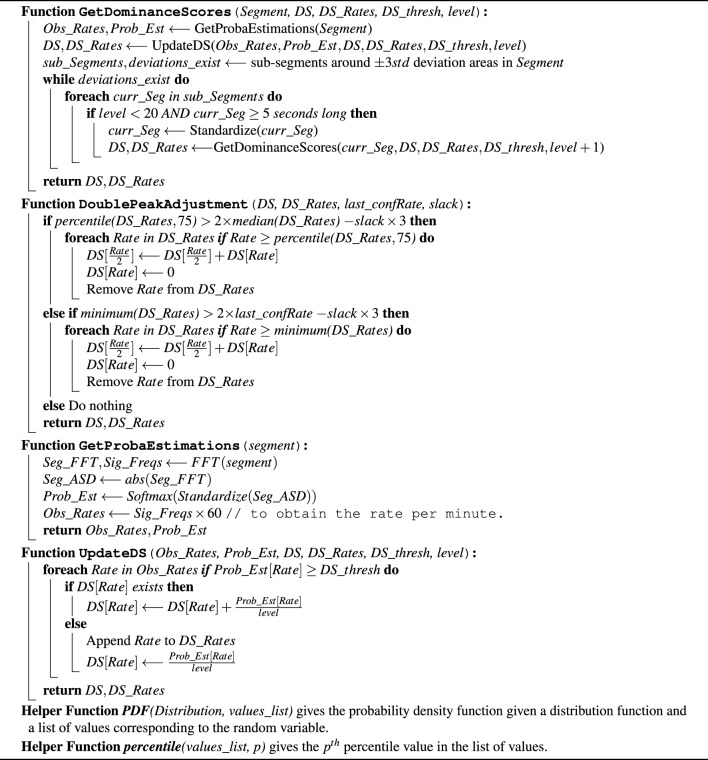
EWMA-driven rate estimation Algorithm, Part 2: Accompanying functions.

#### EWMA-driven rate estimation

Given the dominance scores observed in a processed segment at time *t*, the exponentially weighted moving average (EWMA) value at each possible rate can be calculated, from which an estimated rate-band at time *t* can be inferred. When $t=1$, $\text {EWMA}_{t-1}$ was set to be equal to a probability density function defined by a normal distribution over the possible rate values, parametrized by mean $\mu _m$ and standard deviation $\sigma _m$. $\mu _m$ and $\sigma _m$ were set to 80 and 20 when the measurement task *m* was ‘pulse’ rate, and to 14 and 4 when ’respiratory’ rate was the measurement task, respectively. In each of the two measurement cases, a spread allowance $slack_m$ was defined. This slack was used to determine whether a rate estimate is considered confident or not, by comparing $slack_m$ to the distance from the median to the 25th and 75th percentiles of the rates that have $\text {EWMA}_{t}$ values above 0.1. A distribution of rate values spreading significantly beyond the slack (in both directions from the median) is considered to result in an unconfident rate estimate. The lower the value of the slack the more strict it is to be confident about an estimation, and vice versa.

In the case of pulse rate measurement, a conditional adjustment to the dominance scores was required in order to account for the effect of the double peaks that often occur in the PPG waveform. This was done by evaluating the rate values residing above the 75th percentile against the median value, as well as evaluating the minimum value against the last confident rate estimation, if a predominant double peak effect was evident, the dominance scores associated with those outlier rates were halved. This method is detailed in function *DoublePeakAdjustment* shown in Algorithm 2.

A rate-value estimate $RV\_Est$ was then set to be equal to the rate associated with the maximum dominance score present in the window segment, as defined in Eq. (1) below:

1 $$\begin{aligned} RV\_Est \longleftarrow \arg \max _{Rate} DS \end{aligned}$$

The dominance scores were translated from per-rate estimates to per-rate-band estimates $Curr\_E_t$, where each band is represented by its center *Rate*. This was done by summing over the dominance scores *DS* associated with the rates falling within the variation range bounds defined by [$Rate - \lfloor {\frac{slack_{m}}{2}}\rfloor$, $Rate +\lceil {\frac{slack_{m}}{2}}\rceil$], where $slack_m$ was equal to 5 for pulse rate ($m=$‘*pulse*’) and 3 for respiratory rate ($m=$ ‘*respiratory‘*’). Equation (2) shows the mathematical representation of $Curr\_E_t$:

2 $$\begin{aligned} Curr\_E_t[Rate] \longleftarrow {\left\{ \begin{array}{ll} \sum _{r=Rate - \lfloor {\frac{slack_{m}}{2}}\rfloor }^{Rate + \lceil {\frac{slack_{m}}{2}}\rceil } DS[r] & \textit{ , if } Rate \in DS\_Rates, \\ 0 & \textit{ , otherwise}. \end{array}\right. }\ , \text { for all } Rate \in Rate\_Range_{m}\; \end{aligned}$$

where *DS*[*r*] is the dominance score associated with rate *r*, and $Rate\_Range_{m}$ is a list of all rate values within the range defined per measurement task *m*; so that $Rate\_Range_{m} = [0,1,..., 240]$ when $m=$‘*pulse*’, and $Rate\_Range_{m} = [0,1,..., 60]$ when $m=$*‘respiratory‘*.

The choice of the variation range was made based on the acceptable error allowance in clinical settings, the acceptable error in pulse rate measurement is $\pm \,5$ BPM (ANSI/AAMI 2002)^11,12^ while the error allowance for respiratory rate was chosen to be $\pm\, 3$ BrPM based on the maximum error margin produced by the Capnostream™35, although an error of $\pm\, 5$ BrPM is considered clinically acceptable in many studies^26–28^. The selected bound ranges allow for sufficient measurement variations and reasonable specificity while leaving room for acceptable errors outside the bounds, that is, an access room for clinically acceptable error of $\pm\, 2.5$ BPM in the case of pulse rate estimation, and $\pm\, 1.5$ BrPM in the case of respiratory rate estimation.

The calculation of per-rate-band EWMA values at time *t* is shown in Eq. (3) below:

3 $$\begin{aligned} \text {EWMA}_{t}[Rate] \longleftarrow \alpha \times Curr\_E_t[Rate] + (1-\alpha ) \times \text {EWMA}_{t-1}[Rate] , \text { for all } Rate \in Rate\_Range_{m} \end{aligned}$$

where $Curr\_E_t[Rate]$ is defined in Eq. (2), and $\alpha$ was set to 0.3 as it was found in prior trials to produce superior results compared to 0.7 or 0.5. $\alpha = 0.3$ means that a score at time *t* has 30% influence on the resulting $\text {EWMA}_{t}$, and this influence stays above 2.5% for the following 7 timesteps, due to the exponential decay over time. A timestep is equal to the processing sliding window step which was set to 1 s in this work.

The rate-band estimate at time *t* ($RB_t$) was defined as shown in Eq. (4):

4 $$\begin{aligned} RB_t \longleftarrow {\left\{ \begin{array}{ll} \arg \max _{Rate} \text {EWMA}_{t}[Rate] & \textit{ ,if } t = 1, \\ \min _{Rate}|RB_{t-1} - Rate| & \textit{ ,otherwise}. \end{array}\right. }\ , \text { for all } Rate \in H\_Rates\; \end{aligned}$$

where $H\_Rates$ is a list of rates which correspond to an $EWMA_t$ > *thresh*, and *thresh* = 0.1. Given the EWMA scores at time *t* ($\text {EWMA}_{t}$), the rate-band estimate when $t=1$ was set to be equal to the rate-band that produces the maximum of the $\text {EWMA}_{t}$ scores. While in the case of $t\ne 1$, the estimated rate-band was set to be equal to the rate-band closest to the previously estimated band ($RB_{t}$), provided that its $\text {EWMA}_{t}$ score was above *thresh*. This was done to reduce high variance in the estimations and maintain gradual and more biologically appropriate fluctuations over time.

The estimated rate value at time t ($RV_{t}$), represented by the rate associated with the maximum dominance score, was then examined against the estimated rate-band ($RB_{t}$) and was produced only if it laid within the estimated rate-band range.

All of the aforementioned steps are detailed in pseudocode shown in function *GetRateEstimation* in Algorithm 1

#### Code availability

Code corresponding to the functions in Algorithm 1 and 2 can be found in the following link (written in Python 3.9.13):

https://doi.org/10.5281/zenodo.13169839

### Evaluation measures

Root mean squared error (RMSE), mean absolute error (MAE), and error standard deviation (STD) were the main performance metrics used to evaluate the accuracy of the rate estimates. When the estimations were produced in the form of rate-bands, those metrics were calculated with respect to the bounds of those ranges. For example, a respiratory rate estimate of [11, 14] BrPM would have an error of 0 if the ground truth laid within this range, and the error would be equal to the distance from the closest bound if it laid beyond the range, for instance, the absolute error would be 1 if the ground truth was 15 BrPM.

The percentage of time an output was produced by the estimation framework was also calculated, this equates to how much of the total time a confident estimate (according to the conditions described in the previous section) was given since only confident estimates were allowed to be produced as output. Furthermore, the percentage of produced output estimates that lay within the clinically acceptable error distance from the ground truth was calculated, this corresponded to $\pm\, 5$ BPM in the case of pulse rate and $\pm\, 3$ BrPM in the case of respiratory rate. When producing rate-bands, these distances were calculated relative to the estimated rate-bands, which when their built-in errors are excluded would leave $\pm\, 2.5$ BPM and $\pm \,1.5$ BrPM, respectively.

The agreement between the produced estimations and the ground truth values was further analyzed using the Bland–Altman method, where the mean difference (bias) and the 95% limits of agreement (LoA) are calculated across the paired measurements. Furthermore, the components-of-variance technique was used to account for the effect of subject-dependant observations and intersubject variance when calculating the LoAs^29^.

## Results and discussion

**Fig. 5:**
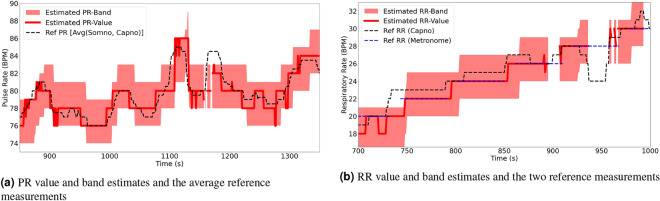
Examples of the produced estimation values and bands as well as the reference measurements when estimating (**a**) pulse rate and (**b**) respiratory rate.

Figure 5a,b show two random examples of the output estimates obtained from two participants. Figure 5a illustrates the PR band and value estimates generated in a fragment of Participant 10’s session, while Fig. 5b gives an example segment of RR band and value estimates belonging to Participant 11, when using both Acc and PPG signals.

### Pulse rate estimation from PPG signals

**Table 1:**
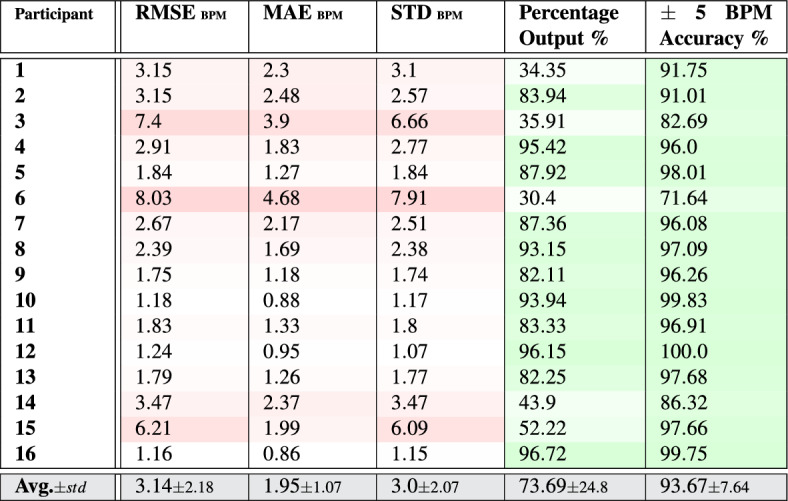
Pulse rate-**value** estimation results per participant and overall with respect to the average of the ground truth measurements given by the two reference systems.

**Fig. 6:**
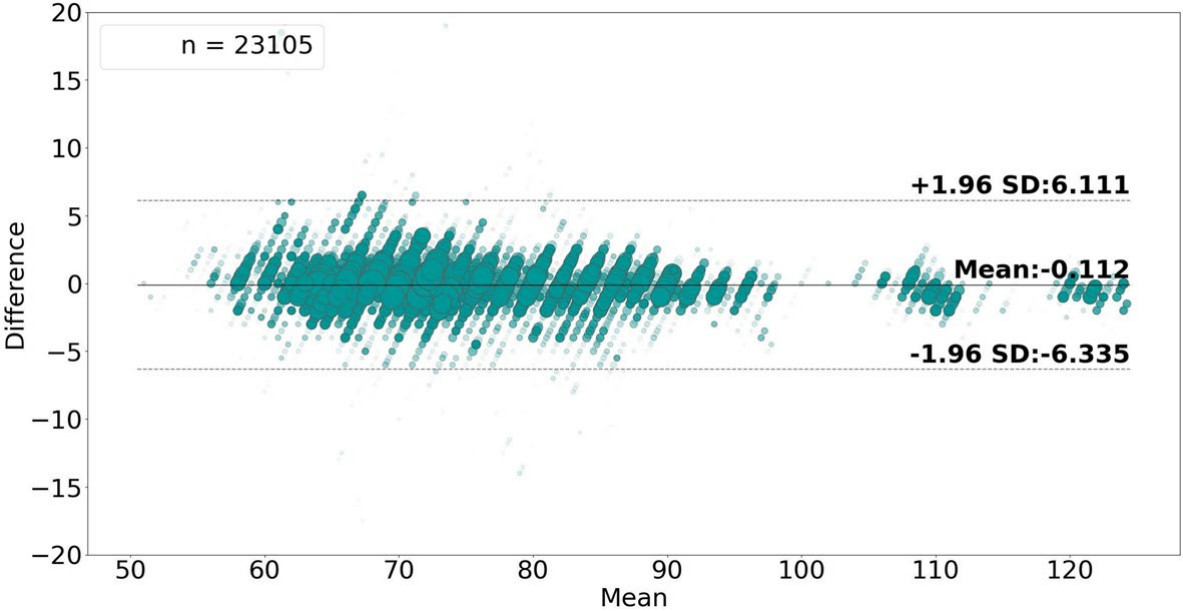
Bland–Altman plot analyzing the agreement between the average ground truth values and the rate-**value** pulse rate estimates. The size and opacity of the dots decrease in proportion to the decreased density of data points.

Tables 1 and 2 list the error measurements when estimating pulse rate values and bands using the PPG signals, respectively. Those measurements were calculated with respect to the average ground truth values obtained from the two reference systems (SOMNOscreen™ plus and Capnostream™35). This was done since both systems produced very close pulse rate measurements throughout the experiments (their root mean squared deviation was $1.07\pm 0.5$). Furthermore, averaging the measurements provided by both systems can result in a ground truth pulse rate measurement closer to the actual true rate than any of them individually.

Observing the tables reveals that the proposed rate estimation framework was able to achieve acceptable errors overall. The average RMSE was $3.14\pm 2.18$ when estimating rate values and $2.25\pm 2.41$ when estimating rate bands, with most of the subjects’ RMSE values falling below 3.5 in both cases except for subjects 3, 6, and 15 which had notably higher error values. The average MAE and STD were $1.95\pm 1.07$ and $3.0\pm 2.07$ in the case of rate-values, and $0.81\pm 1.03$ and $2.07\pm 2.2$ in the case of rate-bands, respectively, with the same subjects driving most of the overall errors.

The tables also list the percentages of time a rate estimation was confident and therefore given as output, which was about $73.69\pm 24.8$ for rate-value estimations, and $78.05\pm 22.71$% for rate-band estimations, each averaged over all subjects, with wide variations across the subjects ranging between 30.4 and 97.6%, the minimum of which belonged to subject 6. The percentage outputs were generally lower when estimating rate values than when estimating rate bands since producing the rate value was under an additional condition; that is, the condition of laying within the associated EWMA-driven rate-band estimation.

Out of the outputted estimates, 93.67 $\pm\, 7.64$% of them were $\pm \,5$ BPM away from the reference average ground truth measurements, in the case of rate-value estimates, and 92.6 ± 8.41% of them were, at most, $\pm\, 2.5$ BPM away in the case of rate-band estimates (which had an internal range of $\pm\, 2.5$ BPM), with a minimum accuracy for participant 6 in both cases and a maximum of $\approx$ 100% for participant 12 and 16 who seem to have produced almost ideal results using this framework. Overall, rate-value estimation seems to produce higher accuracies compared to rate-band estimation as a result of being more strict, as shown in the percentage of outputs.

**Fig. 7:**
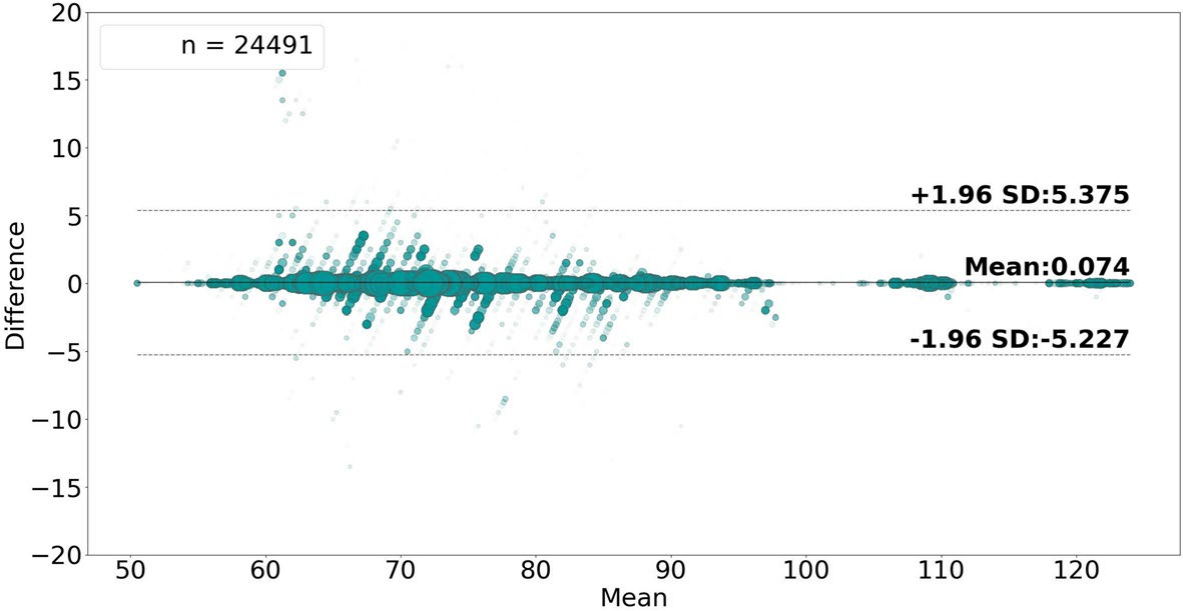
Bland–Altman plot analyzing the agreement between the average ground truth values and the rate-**band** pulse rate estimates. The size and opacity of the dots decrease in proportion to the decreased density of data points.

Figures 6 and 7 show the high level of agreement between the average ground truth measurements and the generated estimates, with 0.112 bias and lower and upper LoA values of − 6.335 and 6.111, in the case of estimating rate-values, and with 0.074 bias and lower and upper LoA values of − 5.227 and 5.375, in the case of rate-band estimation.

**Table 2:**
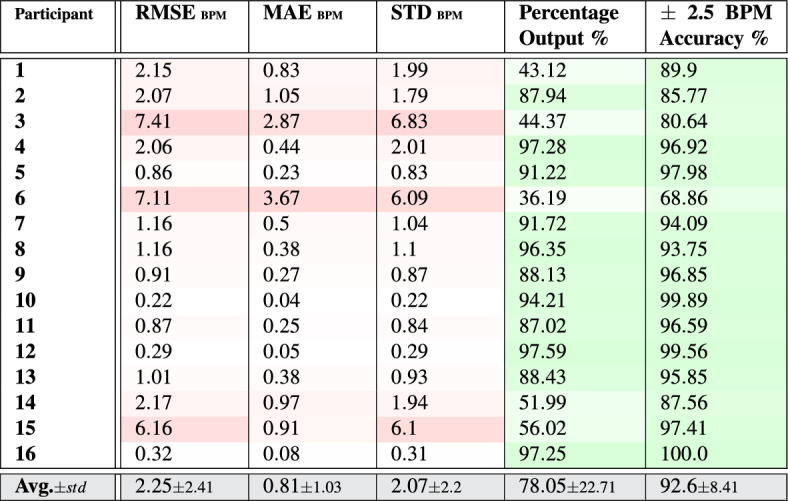
Pulse rate-**band** estimation results per participant and overall with respect to the average of the ground truth measurements given by the two reference systems.

### Respiratory rate estimation from PPG signals

**Table 3:**
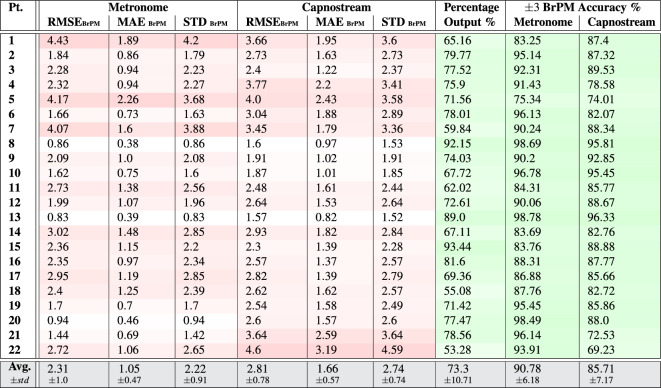
Per participant and overall results of respiratory rate-**value** estimation using **PPG** signals with respect to the visual metronome and the Capnostream™35 reference system.

**Fig. 8:**
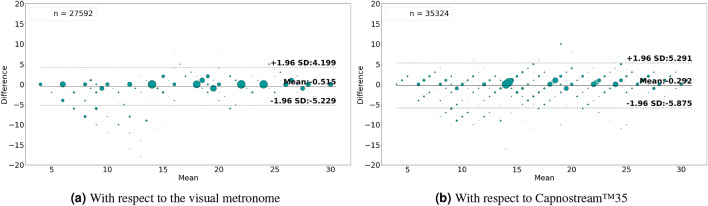
Bland–Altman plots analyzing the agreement between the respiratory rate-**value** estimates and (**a**) the visual metronome settings, or (**b**) the Capnostream™35 measurements, when using **PPG** signals. The size and opacity of the dots decrease in proportion to the decreased density of data points

The respiratory rate estimation results in Tables 3 and 4 show that the proposed framework using PPG signals was able to produce estimates within a reasonable amount of error that was generally maintained below 3 BrPM in most subjects, in both rate-value and rate-band estimates, except for participants 1, 5, 7, 21 and 22 who had an error beyond 3 BrPM in at least one of the measures.

The average RMSE, MAE, and STD values reveal that the proposed framework had less error with respect to the visual metronome (RMSE = $2.31\pm 1.0$, MAE = $1.05\pm 0.47$, and STD = $2.22\pm 0.91$ when estimating rate values, and RMSE= $1.98\pm 0.96$, MAE = $0.7\pm 0.45$, and STD = $1.84\pm 0.86$ when estimating rate bands) compared to its error with respect to the Capnostream™35 (RMSE = $2.81\pm 0.78$, MAE = $1.66\pm 0.57$, and STD = $2.74\pm 0.74$ in the case of rate-value estimations, and RMSE = $2.22\pm 0.72$, MAE = $0.96\pm 0.51$, and STD = $1.99\pm 0.56$ in the case of rate-band estimations). This can be due to the relatively rapid adaptation capabilities of the proposed framework to clear sudden changes in the respiratory rate, another reason could be due to the oral/nasal cannula’s temporary mispositioning before being re-adjusted, causing slight drifts in the reference measurements.

The tables also show that there was a confident output estimation at least 60% of the time in the case of rate-band estimations and 53% when estimating rate values (both belonging to participant 22), with an average of $77.76\pm 9.21$ and $73.3\pm 10.71$ across all participants, respective to the two cases, with the rate-value estimations having less percentage outputs compared to the rate-band estimations (agreeing with the observation discussed in the PR estimation results). Moreover, when estimating rate values, an average of $90.78\pm 6.18$ and $85.71\pm 7.17$ of the produced output estimates had $\pm\, 3$ BrPM accuracy with respect to the visual metronome and the Capnostream™35, while the minimum accuracy among the participants was 75.34% and 69.23%, respectively. In the case of rate-band estimations, an average of $87.9\pm 6.3$ and $80.4\pm 9.74$ of the produced output bands estimates had $\pm \,1.5$ BrPM (excluding the internal $\pm\, 1.5$ BrPM range) accuracy with respect to the visual metronome and the Capnostream™35, while the minimum accuracy among the participants was 73% and 53.23%, respectively. These accuracy results show that the rate-value estimations are more accurate while being reserved as shown in the percentage of outputs, agreeing with the same observation discussed in the PR estimation results.

**Fig. 9:**
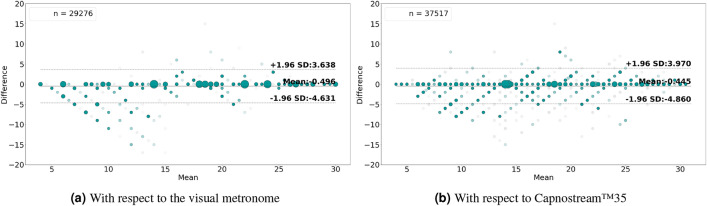
Bland–Altman plots analyzing the agreement between the respiratory rate-**band** estimates and (**a**) the visual metronome settings, or (**b**) the Capnostream™35 measurements, when using **PPG** signals. The size and opacity of the dots decrease in proportion to the decreased density of data points

In terms of the Bland–Altman analysis, Fig. 8a,b show that the proposed framework given PPG signals was able to achieve a mean bias of − 0.515 with LoAs of [− 5.229, 4.199] with respect to the visual metronome, and a mean bias of − 0.292 with LoAs of [− 5.875, 5.291] with reference to the Capnostream™35 measurements, when using the estimated rate values. While Fig. 9a,b show the rate-band estimates’ agreement giving a mean bias of − 0.496 with LoAs of [− 4.631, 3.638] with respect to the visual metronome and a mean bias of − 0.445 with LoAs of [− 4.86, 3.97] with reference to the Capnostream™35 measurements.

**Table 4:**
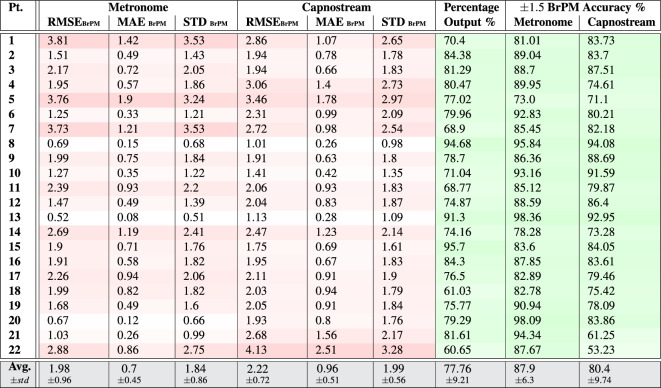
Per participant and overall results of respiratory rate-**band** estimation using **PPG** signals with respect to the visual metronome and the Capnostream™35 reference system.

### Respiratory rate estimation from Acc signals

**Table 5:**
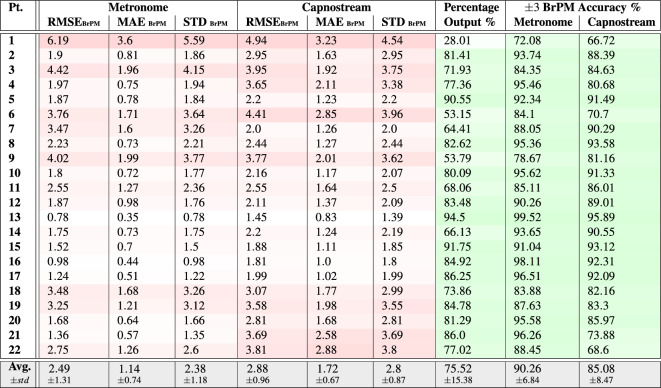
Per participant and overall results of respiratory rate-**value** estimation using **Acc** signals.

**Fig. 10:**
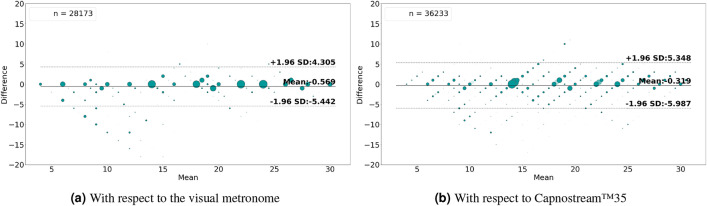
Bland-Altman plots analyzing the agreement between the respiratory rate-**value** estimations and (**a**) the visual metronome settings, or (**b**) the Capnostream™35 measurements, when using **Acc** signals. The size and opacity of the dots decrease in proportion to the decreased density of data points

Providing the proposed framework with Acc signals for respiratory rate estimation resulted in accuracies that, although inferior to when using the PPG signals, can be considered generally acceptable. Table 5 listing the rate-value estimation results show that the average RMSE, MAE, and STD when calculated in relation to the visual metronome were $2.49\pm 1.31$, $1.14\pm 0.74$, and $2.38\pm 1.18$, respectively, and those measures were slightly higher when calculated with respect to the Capnostream™35, being equal to $2.88\pm 0.96$, $1.72\pm 0.67$, and $2.8\pm 0.87$, respectively. While Table 6 listing the rate-band estimation results show that the average RMSE, MAE, and STD when calculated relative to the visual metronome were $2.18\pm 1.44$, $0.81\pm 0.82$, and $2.01\pm 1.21$, and equal to $2.36\pm 1.04$, $1.03\pm 0.66$, and $2.1\pm 0.85$ when calculated with respect to the Capnostream™35, respectively. The estimation error measures in both cases were generally below 4 BrPM for most participants, except for subjects 1, 3, 6, and 9 who suffered from relatively higher errors.

The percentage of output estimates was generally high across most participants, ranging between 53.15 and 94.5% when estimating rate values and 59.49% to 95.25% when estimating rate bands, respectively, with the exception of Participant 1 who had a low percentage output in both modes, which could be due to accessive body movements that might have affected the consistency of the Acc readings, and therefore reduced the amount of time a confident estimation was produced.

Among the outputted estimates, when estimating rate values, the overall $\pm\,\, 3$ BrPM accuracy was $90.26\pm 6.84$% and $85.08\pm 8.47$% with reference to the visual metronome and the Capnostream™35, respectively, with a minimum accuracy of 72.08% and 66.72% given by participant 1. When estimating rate bands, the overall $\pm 1.5$ BrPM accuracy (excluding the internal $\pm\, 1.5$ BrPM band range) was $87.6\pm 10.03$% and $80.12\pm 10.96$% with respect to the visual metronome and the Capnostream™35, respectively. The Tables show that the minimum accuracies in both cases belonged to Participant 1.

**Fig. 11:**
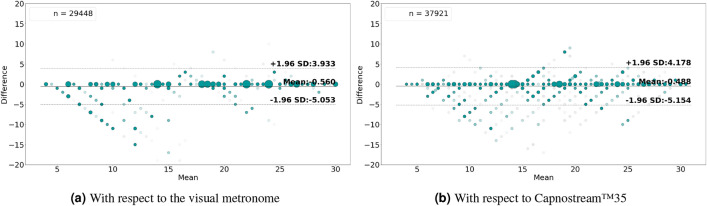
Bland–Altman plots analyzing the agreement between the respiratory rate-band estimates and (**a**) the visual metronome settings, or (**b**) the Capnostream™35 measurements, when using Acc signals. The size and opacity of the dots decrease in proportion to the decreased density of data points

The Bland–Altman analysis presented in Fig. 10a,b for rate-value estimates and Fig. 11a,b for rate-band estimations show that the proposed framework fed with Acc signals achieved a mean bias of − 0.569 with LoAs of [$-5.442$, 4.305] and a mean bias of − 0.56 with LoAs of [− 5.053, 3.933], respectively, with respect to the visual metronome and a mean bias of − 0.319 with LoAs of [− 5.987, 5.348] and a mean bias of − 0.488 with LoAs of [− 5.154, 4.178], respectively, with reference to the Capnostream™35 measurements.

**Table 6:**
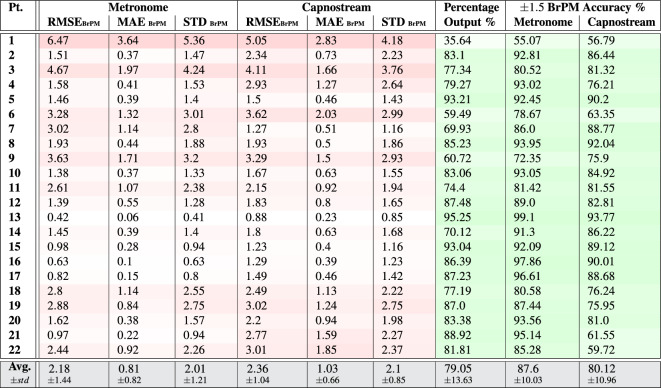
Per participant and overall results of respiratory rate-**band** estimation using **Acc** signals.

### Respiratory rate estimation from PPG and Acc signals

**Table 7:**
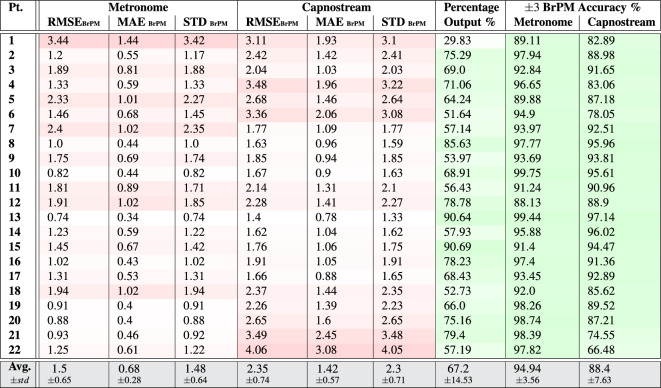
Per participant and overall results of respiratory rate-**value** estimation using both** PPG and Acc** signals.

**Fig. 12:**
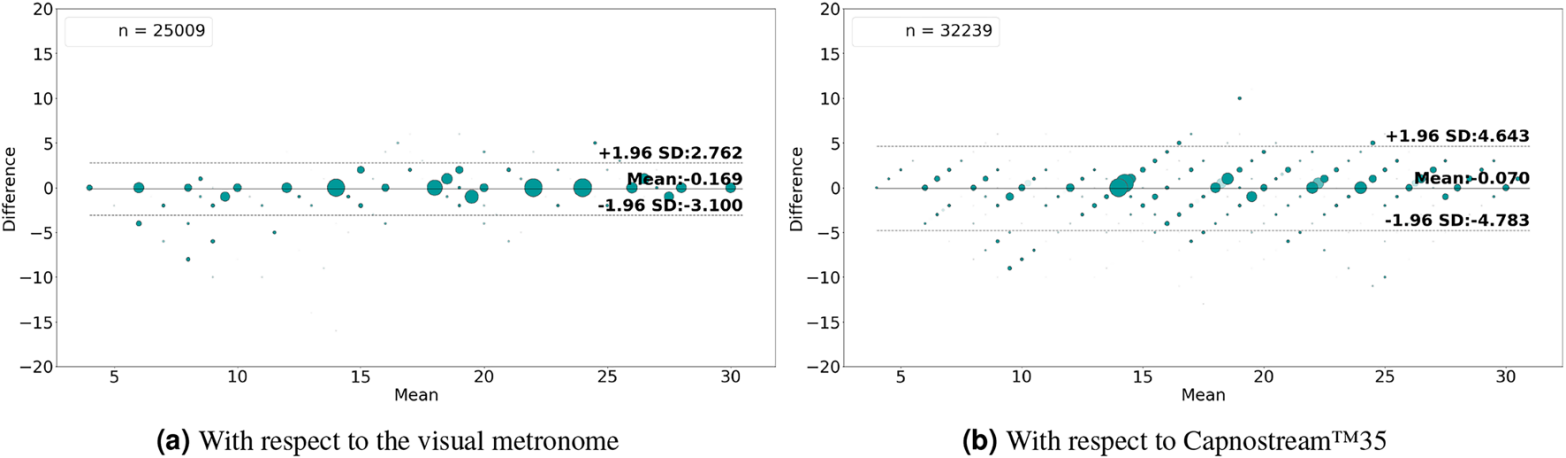
Bland–Altman plots analyzing the agreement between the respiratory rate-**value** estimates and (**a**) the visual metronome settings, or (**b**) the Capnostream™35 measurements, when using both **PPG and Acc** signals. The size and opacity of the dots decrease in proportion to the decreased density of data points

Tables 7 and 8 show that, overall, the respiratory rate estimation errors when using the proposed framework using as inputs both PPG and Acc signals were almost entirely maintained below 3.5 BrPM in all measures and across most participants, except for participant 22 in the case of rate-value estimation when evaluated against the Capnostream™35, with RMSE and STD values approaching $\approx$ 4 BrPM. These results surpass the estimation performance of using either modality separately.

When estimating rate values, the average RMSE, MAE, and STD were $1.5\pm 0.65$, $0.68\pm 0.28$, and $1.48\pm 0.64$ when calculated in reference to the visual metronome, respectively, and the respective measures were $2.35\pm 0.74$, $1.42\pm 0.57$, and $2.3\pm 0.71$ when calculated with respect to the Capnostream™35. In the case of rate-band estimation, the three measures when calculated in reference to the visual metronome were $1.09\pm 0.69$, $0.31\pm 0.27$, and $1.04\pm 0.64$, respectively, and $1.45\pm 0.62$, $0.54\pm 0.38$, and $1.34\pm 0.52$ when calculated against the Capnostream™35.

The percentages of output estimates were mostly between $\approx 51$% and $\approx 93$% across both the rate-value and rate-band cases, with Participant 1 suffering from the lowest percentage of confident output estimates at 29.83% when estimating rate values and 36.93% when estimating rate bands, which can be explained by the same reasoning presented in the previous subsection due to the use of Acc signals. Given the device placement on the neck, body movements without moving the head could result in motion artifacts that are present more strongly in the Acc signals than in the PPG signals, and this is evident in the difference between the output percentages of the same participant in Tables 3 & 4 and 5 & 6. In those cases, the motion artifacts can lower the overall confidence and prevent the generation of output rate estimates. This effect can be mitigated by adaptively blocking the use of a modality when a relevant type of motion artifact is detected.

Table 7 also shows that an average of $94.94.07\pm 3.56$% and $88.4\pm 7.63$% of the outputted rate estimates were at most $\pm 3$ BrPM away from the metronome rate and Capnostream™35, respectively. While $93.07\pm 4.46$% and $87.86\pm 8.2$% of them were at most $\pm 1.5$ BrPM (outside of the internal $\pm 1.5$ BrPM band range) away from the metronome rate and Capnostream™35, respectively, as shown in Table 8.

**Fig. 13:**
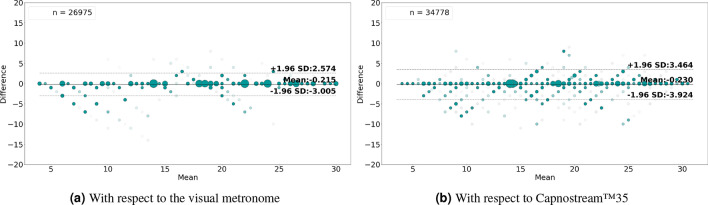
Bland-Altman plots analyzing the agreement between the respiratory rate-**band** estimates and (**a**) the visual metronome settings, or (**b**) the Capnostream™35 measurements, when using both **PPG and Acc** signals. The size and opacity of the dots decrease in proportion to the decreased density of data points.

Figures 12a & 13a and Figures 12b & 13b show the Bland-Altman analysis plots when evaluating the rate estimates with reference to the visual metronome and the Capnostream™35, in the case of rate-value and rate-band estimations, respectively. When evaluated against the visual metronome, the framework achieved a mean bias of − 0.169 with LoAs of [− 3.100, 2.762] when estimating rate values, and a mean bias of − 0.215 with LoAs of [− 3.005, 2.574] when estimating rate bands. While evaluating with respect to the Capnostream™35 resulted in a mean bias of − 0.07 with LoAs of [− 4.783, 4.643] in the case of rate-value estimation, and a mean bias of − 0.23 with LoAs of [− 3.924, 3.464] in the case of rate-band estimation. Again, the narrower agreement regions show the superiority of using both PPG and Acc signals together for respiratory rate estimation, compared to using each of them separately.

These results, as described in previous sections, show that the respiratory rate estimations using the proposed methodology tended to have lower errors compared to the visual metronome than to the reference Capnostream™35 output, agreeing with the same observations drawn when evaluating the performance when using each modality separately. Furthermore, they also illustrate that the proposed methods can achieve clinically acceptable respiratory rate estimation capabilities when given adequate data.

**Table 8:**
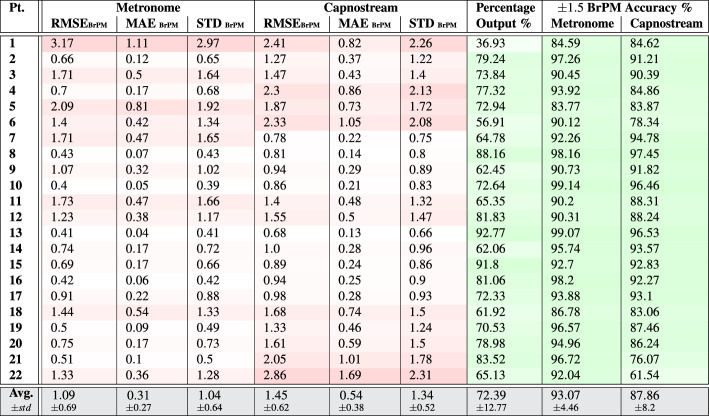
Per participant and overall results of respiratory rate-**band** estimation using both** PPG and Acc** signals.

### Benchmarking with relevent work

Tables 9 and 10 summarize the results obtained from the most relevant latest work, when estimating RR and PR, respectively. Relevance was established by including studies that used data from healthy subjects while at rest, and which reported clear accuracy measures or Bland–Altman analysis.

**Table 9:** RR estimation performance compared to relevant studies.

Paper	Sensor type	Device type	Connectivity	Location	Participants	Duration	Conditions	Range BrPM	Accuracy
Eisenberg et al.^5^	Acoustic (Radical-7)	Not Wearable	Wired	Neck	29 (21 M, 8 F) $23.6\pm 4.4$ Years Old − kg/m^2^	$\approx$ 12 min	Lying supine (controlled)	4-30	$76.8\pm 13$% ($\pm 2$ BrPM)
	PPG (Nellcor™)	Not Wearable	Wired	Finger					$81\pm 11.6$% ($\pm 2$ BrPM)
Doheny et al.^30^	Inertial Sensor (BiostampRC)	Wearable	Wireless	Chest	11 (9 M, 2 F) $47.82\pm 14.14$ Years Old $30.9\pm 5.27$ kg/m^2^	$\approx$ 5 hours	Overnight (uncontrolled)	–	83.6% ($\pm 2$ BrPM)
				Abdomen					80.9% ($\pm 2$ BrPM)
Singh et al.^31^	PPG	Wearable	Wireless	Neck	15 (10 M, 5 F) $27\pm 2$ Years Old $23.8\pm 3.57$ kg/m^2^	$\le 5$ min	Lying supine (controlled)	$\approx$10–17	Bias = 0.43 LoA = (−2.4, 3.3)
Shuzan et al.^32^	PPG*	Not Wearable	Wired	Finger	39 (18 M, 21 F) 29 Years Old (median) 23 kg/m^2^ (median)	$\approx 10$ min	Lying supine (controlled)	5–32	Bias $\approx$ 0 LoA = (−5.16, 5.25)
Romano et al.^33^	Inertial Sensors	Wearable	Wireless	Chest	15 (10 M, 5 F) $26.1\pm 2.3$ Years Old 23.093 kg/m^2^ (mean)	130 s	Lying supine (controlled)	6–30	Bias $\approx$ 0 LoA $\approx \pm 4.5$
This work **	PPG	Wearable	Wireless	Neck	22 (15 M, 7 F) $28\pm 5.26$ Years Old $23.7\pm 3.89$ kg/m^2^	30 min	Lying supine (controlled)	4–30	$85.71\pm 7.17$% ($\pm 3$ BrPM) Bias = −0.29 LoA = (− 5.88, 8.29)
	Acc								$85.08\pm 8.47$% ($\pm 3$ BrPM) Bias = −0.32 LoA = (−5.98, 5.35)
	PPG & Acc								$88.4\pm 7.63$% ($\pm 3$ BrPM) Bias = −0.07 LoA = (−4.78, 4.64)

*VORTAL dataset.

**The accuracies shown correspond to the results when evaluating the estimated rate values against the Capnostream™35.

**Table 10:** PR estimation performance compared to relevant studies.

Paper	Sensor type	Device type	Connectivity	Location	Participants	Duration	Conditions	Range BPM	Accuracy
Sharma et al.^21^	Acoustic	Wearable	Wireless	Neck	29 (- M, - F) − Years Old –kg/m^2^	$\approx$ 5.5 hours	Overnight (uncontrolled)	$\approx$40–160	94.34% ($\pm 10$ BPM) Bias $\approx$ 0 LoA $\approx \pm 7$
Garcia et al.^34^	PPG	Wearable	Wired	Neck	9 (5 M, 4 F) $24\pm 3$ Years Old $22.6\pm 3.7$ kg/m^2^	140 s	Lying supine (controlled)	50–85	Bias = −0.16 LoA = (−4.7 , 4.4)
Singh et al.^31^	PPG	Wearable	Wireless	Neck	15 (10 M, 5 F) $27\pm 2$ Years Old $23.8\pm 3.57$ kg/m^2^	$\le 5$ min	Lying supine (controlled)	40–100	Bias = −0.67 LoA = (−4.6 , 3.3)
Romano et al.^33^	Inertial Sensors	Wearable	Wireless	Chest	15 (10 M, 5 F) $26.1\pm 2.3$ Years Old 23.093 kg/m^2^ (mean)	130 s	Lying supine (controlled)	$\approx$50–100	Bias $=0.1$ LoA $=\pm 2$
Butkow et al.^35^	Acoustic	Wearable	Wired	In-ear	15 (8 M, 7 F) − Years Old − kg/m^2^	5 min	Lying at rest (controlled)	45–97	Bias = 0.66 LoA = (−5.97 , 7.29)
This work*	PPG	Wearable	Wireless	Neck	16 (9 M, 7 F) $29\pm 6.3$ Years Old $23.52\pm 3.7$ kg/m^2^	26–35 min	Lying supine (controlled)	45–124	$93.67\pm 7.64$% ($\pm 5$ BPM*) Bias = $-0.112$ LoA = (−6.34 , 6.11)

*The accuracies shown correspond to the results produced by the rate-value estimations.

Referring to the RR estimation studies listed in Table 9, it can be observed that the proposed methodology provided competitive estimation performance over a wide range of rates.

Eisenberg et al.^5^ validated the performance of two commercial devices in a controlled study, namely, Radical-7 (Radical-7+RAS-125 Version C, Masimo Corporation) and Nellcor™ Adult Respiratory Sensor (Medtronic) against Capnostream20p™ (Medtronic) as the ground truth. They reported their $\pm 2$ BrPM estimation accuracies to be $76.8\pm 13$ and $81\pm 11.6$, respectively, for breathing rates between 4 and 30 BrPM.

Doheny et al.^30^ studied the use of BiostampRC inertial sensors placed on the chest and the abdomen in an overnight study, and reported $\pm 2$ BrPM accuracies when participants were lying supine equal to 83.6% and 80.9%, respectively. In their work, RR was inferred using peak detection applied to the accelerometer and pitch signals using fixed minimum time between peaks and adaptive thresholding based on signal amplitude and sleep position, after being filtered using an 8th-order low-pass filter [0.5 Hz] and notch filter to remove frequencies below 0.05 Hz. Interestingly, they found that lying on the side resulted in reduced errors compared to lying supine.

Singh et al.^31^ presented high RR estimation agreement (Bias of 0.43 and 95% LoA of − 2.4 and 3.3 BrPM) with the ground truth system SOMNOscreen (SOMNOmedics) when using neck PPG signals from subjects lying supine for up to 5 min. RR estimates were produced by obtaining the maximum energy in the time-frequency domain using MATLAB’s *fridge* function after the PPG signals were filtered using 4th-order Chebyshev [0.1–0.5 Hz] and 3rd-order 15s-window Savitzky–Golay filters. Although they reported high-accuracy estimations, the validated range was relatively small ($\approx$ 10–17 BrPM). Furthermore, the normalization and signal processing approach was performed over whole recordings rather than a window-based approach, which makes it weakly transferable to real-time estimation tasks.

Shuzan et al.^32^ used finger PPG signals which were passed through a 6th-order IIR filter [25 Hz], followed by a variational mode decomposition (VMD) to further remove motion artifacts by reconstructing the PPG signals using the first 4 modes. 11 time-domain features were used to train and optimize a gaussian process regression model that estimated RR with an acceptable agreement (Bias of near zero and 95% LoA of − 5.16 and 5.25 BrPM) with their ground truth measurements obtained from Ultima Dual Airflow differential pressure transducer (Braebon Medical Corporation).

Romano et al.^33^ explored the use of 5 inertial sensors distributed across the chest area, and evaluated the RR estimation capabilities as subjects performed different activities, which included lying supine for 130 s. Their methodology comprised signal quality evaluation in order to determine which sensor carried the most information at any given time, which was defined by the highest normalized power density (nPSD) amplitude. Signals were filtered using 1st-order Butterworth band-pass filter [0.05–1 Hz], and RR was then calculated based on the time duration between consecutive peaks. The degree of agreement with the rate obtained from their ground truth system Zephyr BioHarness 3.0 (Medtronic) when lying supine was not clearly stated, however, by observing the Bland-Altman plot in the paper (Fig. 7 in^33^), a bias of near zero with 95% LoA of $\approx \pm 4.5$ was achieved.

As for PR estimation, Table 10 shows that the proposed methodology, again, provided competitive estimation performance, compared to the relevant listed studies.

Sharma et al.^21^ proposed a methodology for PR estimation using a neck-located acoustic sensor, which was evaluated in an overnight uncontrolled study. The method utilized the Hilbert energy envelope along with adaptive thresholding in order to infer PR. They reported an $\pm 10$ BPM accuracy of 97.34% , bias of near zero and 95% LoA of $\pm 7$ with respect to ground truth measurements obtained by SOMNOscreen™ plus (SOMNOmedics). Those measures were evaluated within the range $\approx$ 40–160 BPM.

Garcia et al.^34^ investigated—for the first time—the use of neck PPG for PR estimation in a controlled study. They applied a 4th-order high-pass Butterworth filter [0.7 Hz], and then used the time between peaks along with adaptive thresholding to infer the PR. This method was able to achieve a bias of − 0.16 and 95% LoA of − 4.7 and 4.4, with ground truth measurements obtained from SOMNOscreen™ plus (SOMNOmedics). Although the estimation accuracy was high, the short duration of time per participant (140 s) and the small range of evaluated PR values (50–85 BPM) could limit the generalization of those results. Singh et al.^31^ also investigated the use of neck PPG for PR measurement in their controlled study. They applied a 4th-order Chebyshev filter [0.5–3 Hz], and then used an oscillator-based adaptive notch filter in order to obtain the PR estimates. They achieved a bias of − 0.67 and 95% LoA of − 4.6 and 3.3, within the range 40–100 BPM, compared to the SOMNOscreen™ plus (SOMNOmedics) measurements. Although both Garcia et al.^34^ and Singh et al.^31^ were able to report good estimation accuracies using neck PPG, the PPG sensor was carefully placed away from any veins by using an IR camera to inspect each subject’s neck, which helped ensure better signal quality compared to placing the sensor without inspecting or trying to avoid the veins (which was the case in this work).

Romano et al.^33^ presented their PR estimation capabilities when using the 5 inertial sensors placed on the chest, compared to the ground truth values calculated using the single-lead ECG signal provided by Zephyr BioHarness 3.0 (Medtronic). Similar to their RR estimation methodology mentioned above, signal quality was used to choose the sensor with the highest information content. The signals were band-pass filtered [10–40 Hz] using continuous wavelet transform, then the Hilbert envelope was obtained and filtered using a Butterworth band-pass filter [0.7–3 Hz]. The time between consecutive peaks was used to calculate PR. They reported bias of 0.1 and LoA of $\pm 2$. Although this level of agreement is considered high, the short duration of time per subject (130 s) can reduce the statistical power of this result.

Butkow et al.^35^ presented a novel approach for detecting PR which utilized an in-ear acoustic sensor. The methodology included a 4th-order Butterworth band-pass filter [0.5–50 Hz], followed by short-time Fourier transform in order to compute the spectrogram, which was normalized and then fed to a pre-trained U-Net model which attempted to map acoustic spectrograms to ECG spectrograms (obtained by Zephyr BioHarness 3.0 (Medtronic)). After training, the model’s output was used to reconstruct the time-domain representation (which should represent the ECG waveform) using the Griffin-Lim algorithm, of which the envelope was obtained using the Hilbert transform, and time between peaks was used to calculate PR. Estimates were then smoothed using a 5-sample moving average. The study incorporated different activities including lying down at rest for 5 min, and the evaluated range was 45–97 BPM. The proposed method achieved a bias of 0.66 and LoA of $-5.97$ and 7.29, with respect to the ground truth PR calculated using Zephyr’s ECG signal.

### Limitations and future work

This study was designed to simulate sleep conditions. However, it lacks evaluation in the presence of snoring, which is likely to be the most frequent artifact present during sleep. It also only evaluates respiratory rate under 30 BrPM, since this was what could be asked from healthy volunteers to simulate. This rate, however, might be below the RR range associated with certain respiratory diseases during sleep. Furthermore, this was a controlled evaluation in healthy volunteers. Hence, the continuation of this work should focus on evaluating, and if needing optimizing the performance of the algorithms, in the real-world setting (i.e. uncontrolled conditions associated with natural sleep where snoring would occur) and with a wide variety of patients, not only healthy subjects.

The absence of the Acc signals for pulse rate estimation is a limitation of this work that prevented the evaluation of the capabilities of the framework when using both modalities. However, based on the respiratory rate results when using PPG and Acc, it is likely that the overall performance would benefit from the addition of the Acc signals. Another noticeable limitation of this framework in multi-modal estimation is the ability to evaluate the type of artifacts imposed in each of the input signals in order to decide if one of those modalities needs to be temporarily ignored, further refinements are needed to address this issue.

Although EWMA is an attractive method for smoothing time-series data and trend estimates due to its simplicity and ease of implementation as well as its memory efficient qualities, its performance highly depends on the appropriate choice of the variable $\alpha$. Therefore, the in-depth analysis of the EWMA-based aggregation approach where the isolated contribution of this method and the performance changes associated with each $\alpha$ value can be of great interest to future studies.

## Conclusion

This paper proposes a novel methodology for pulse rate and respiratory rate estimation using PPG and Acc signals acquired from the neck, where recursive FFT-Based scoring coupled with EWMA-based aggregation was utilized. This work also introduces rate-band estimation that can allow for reduced susceptibility to errors due to temporal fluctuations, while being within clinically acceptable error margins. The proposed framework can be adaptable in terms of the type and number of input signals, as long as those input signals convey periodic changes corresponding to the target rate being estimated.

When estimating pulse rate values using PPG signals, this framework was able to achieve an overall clinically acceptable accuracy of $93.67\pm 7.64$%. Utilizing both PPG and Acc signals for respiratory rate value estimation using this framework generally outperformed using either modality separately, giving an overall clinically appropriate accuracy of $94.94\pm 3.56$% with reference to the guiding visual metronome, and $88.4\pm 7.63$% with respect to the reference device (Capnostream™35). Given that clinically acceptable errors for PR and RR were considered to be $\pm 5$ BPM and $\pm 3$ BrPM, respectively. Although the results in this work are promising, they are not perfect, especially in terms of the percentage of output estimates which averaged $\approx$ 67–79%; adjusting the confidence criteria or the range of the estimation bands, in addition to having a diverse multi-modal input could provide great improvements.

## Data Availability

Data supporting the findings of this study are available from the corresponding author upon reasonable request.
